# Poly[[{μ_2_-5-[(di­methyl­amino)(thioxo)meth­oxy]benzene-1,3-di­carboxyl­ato-κ^4^*O*^1^,*O*^1′^:*O*^3^,*O*^3′^}(μ_2-_4,4′-di­pyridyl­amine-κ^2^*N*^4^:*N*^4′^)cobalt(II)] di­methyl­formamide hemisolvate monohydrate]

**DOI:** 10.1107/S2414314624004899

**Published:** 2024-06-04

**Authors:** Hui-Yu Qin, Bing-Guang Zhang, Qiao-Zhen Sun

**Affiliations:** a Key Laboratory of Catalysis and Materials Sciences of the State Ethnic Affairs Commission & Ministry of Education College of Chemistry and Material Science South-Central Minzu University Wuhan 430074 People’s Republic of China; b School of Materials Science and Engineering Central South University,Changsha 410083 People’s Republic of China; Vienna University of Technology, Austria

**Keywords:** crystal structure, coordination polymer, cobalt, 5-[(di­methyl­amino)­thioxometh­oxy]-1,3-benzene­dicarboxyl­ate, 4,4′-di­pyridyl­amine, hydrogen bonding

## Abstract

In the crystal structure of the title compound, {[Co(C_11_H_9_NSO_5_)(C_10_H_9_N_3_)]0.5C_3_H_7_NO·H_2_O}_*n*_ or {[Co(dmtb)(dpa)]·0.5DMF·H_2_O}_*n*_ (dmtb^2–^ = 5-[(di­meth­yl­amino)­thioxometh­oxy]-1,3-benzene­dicarboxyl­ate and dpa = 4,4′-di­pyridyl­amine), an assembly of periodic [Co(C_11_H_9_NSO_5_)(C_10_H_9_N_3_)]_*n*_ layers extending parallel to the *bc* plane is present. Each layer is constituted by distorted [CoO_4_N_2_] octa­hedra, which are connected through the *μ*_2_-coordination modes of both dmtb^2–^ and dpa ligands. Occupationally disordered water and di­meth­yl­formamide (DMF) solvent mol­ecules are located in the voids of the network to which they are connected through hydrogen-bonding inter­actions.

## Abstract 2

The crystal structure of the title compound shows a layered arrangement parallel to the *bc* plane where [CoO_4_N_2_] octa­hedra are linked by dmtb^2–^ and dpa ligands.

## Graphical abstract

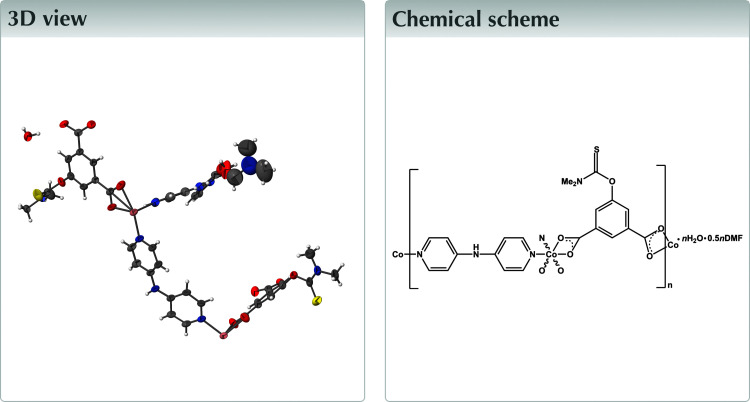

## Structure description

The controllable synthesis of coordination polymers with desired structures is always a challenging subject in crystal engineering (Chung *et al.*, 2023 ▸; Li *et al.*, 2021 ▸; Yang *et al.*, 2021 ▸). In many cases, it is difficult to achieve due to the complex inter­play of different factors and synthesis parameters such as the preferred coordination environment of the central metal atom, the nature of ligand(s), reaction/incorporation of solvents, temperature, metal-to-ligand ratio, pH value, pressure *etc*. (Sun *et al.*, 2016 ▸, 2017 ▸, 2018 ▸; Vornholt *et al.*, 2017 ▸).

According to our previous studies (Gu *et al.*, 2023 ▸; Sun *et al.*, 2019 ▸), the configuration of the secondary ligand can effectively adjust the steric hindrance within the crystal structure. When Zn^2+^ is coordinated by dmtb^2−^ {5-[(di­methyl­amino)­thioxometh­oxy]-1,3-benzene­dicarboxyl­ate} and bipy (4,4′-bi­pyridine), the (di­methyl­amino)­thioxometh­oxy group of the dmtb^2–^ ligand increases the steric hindrance, and a di-periodic, *i.e.* layered, arrangement results. The rigid bipy ligand acts as a pillar in the structural organization (Gu *et al.*, 2023 ▸). In this context and in comparison with the former synthesis, we used the slightly larger Co^2+^ cation and the more flexible 4,4′-di­pyridyl­amine (dpa) ligand for the current study. As a result, the title compound, (**1**), with a likewise layered structural arrangement, was obtained.

**Figure 1:**
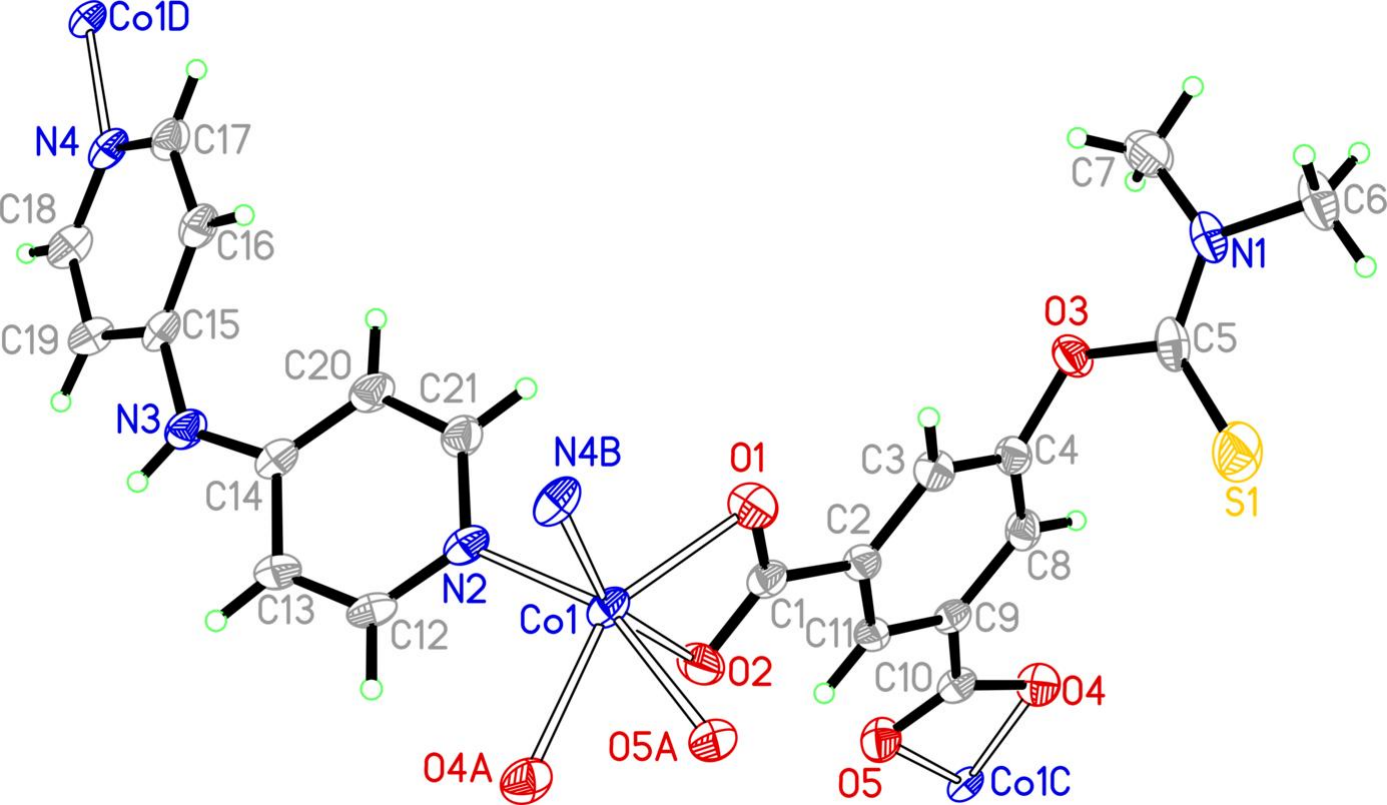
The extended asymmetric unit of (**1**) showing the coordination environment of the Co^2+^ cation. Displacement ellipsoids are drawn at the 30% probability level. The solvent water and DMF mol­ecules are not shown for clarity. [Symmetry codes: (A) −*x* + 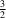, *y* + 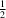, −*z* + 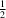; (B) −*x* + 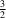, *y* + 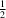, −*z* + 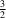; (C) −*x* + 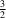, *y* − 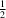, −*z* + 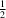; (D) −*x* + 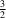, *y* − 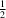, –*z* + 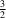.]

**Figure 2:**
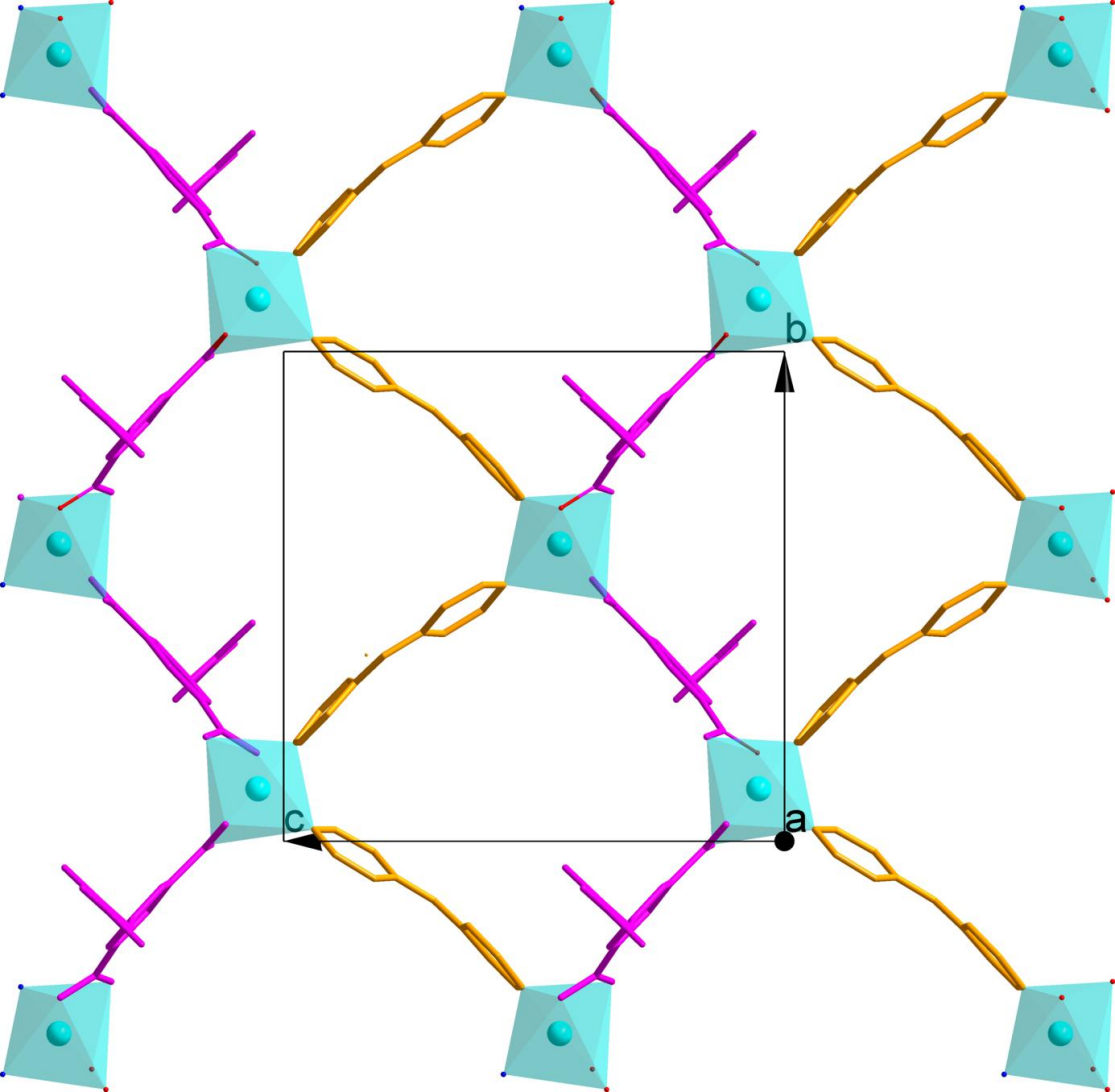
The layered arrangement extending parallel to the *bc* plane in the crystal structure of (**1**).

**Figure 3:**
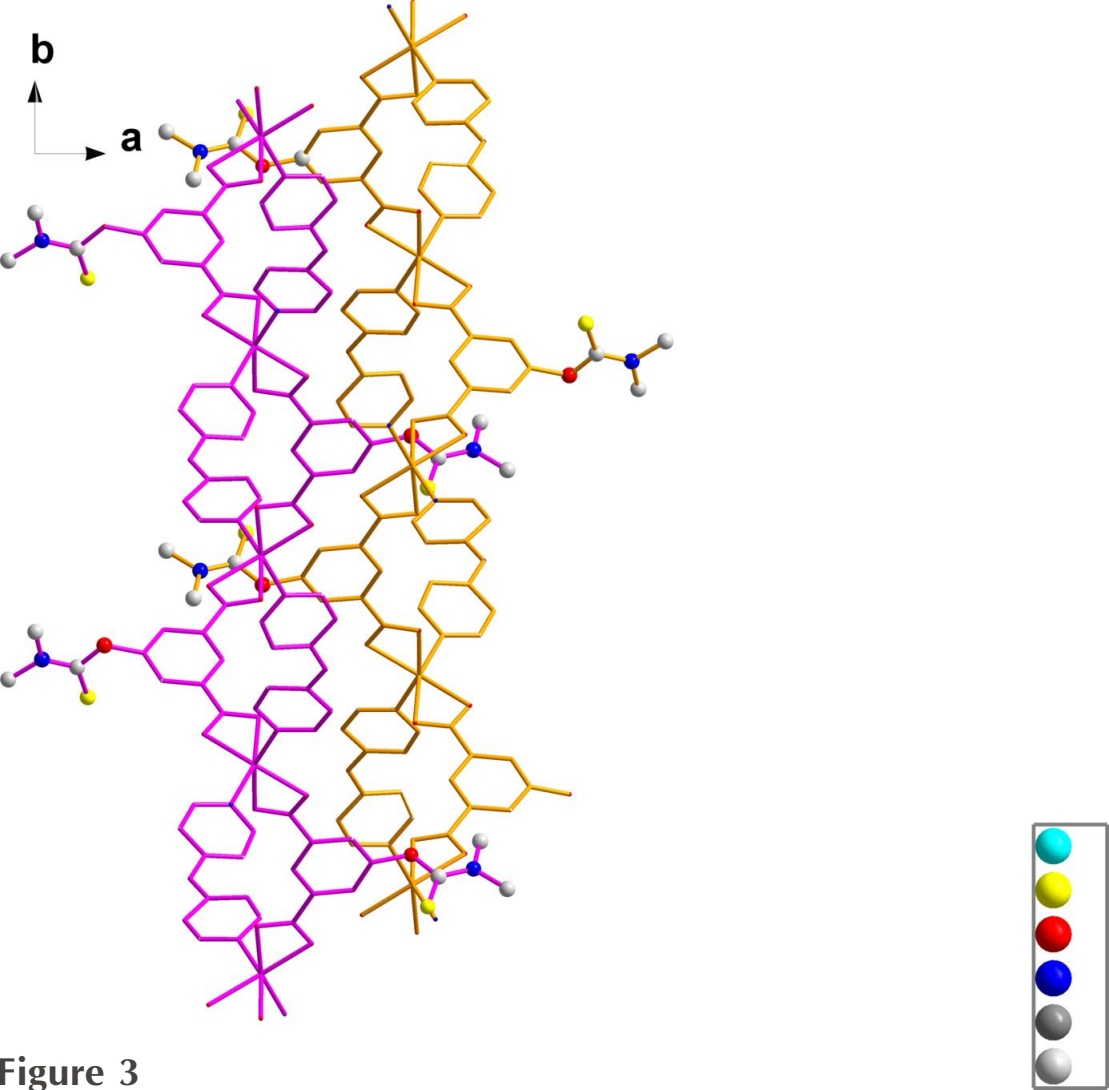
5-(Di­methyl­amino)­thioxometh­oxy moieties of the dmtp^2–^ ligand protruding into an adjacent layer.

**Figure 4:**
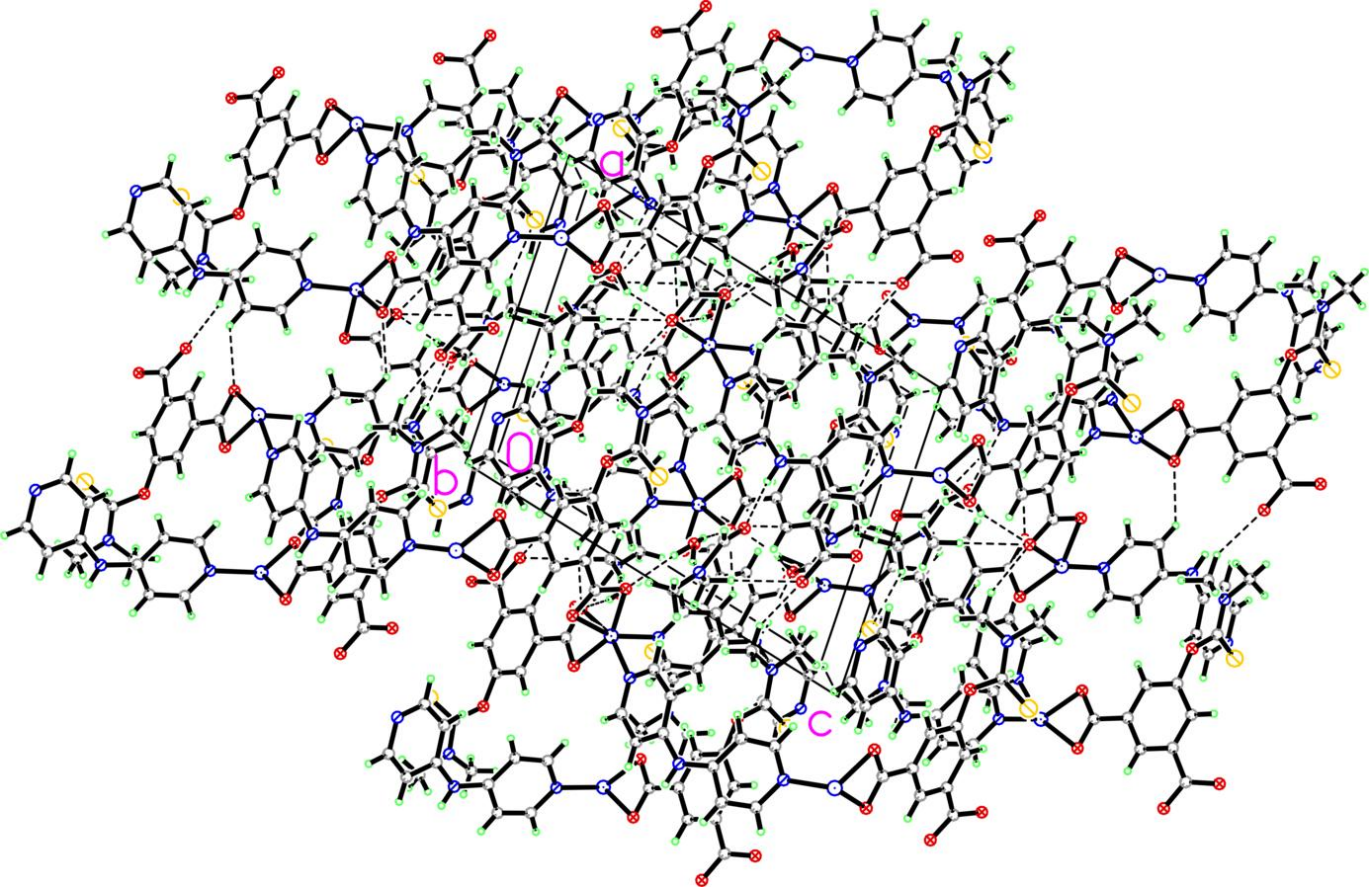
Packing diagram of (**1**), showing hydrogen-bonding inter­actions (dashed lines).

The asymmetric unit of (**1**) (Fig. 1 ▸) comprises one cobalt(II) cation, one dmtb^2−^ anion, one dpa ligand, two occupationally disordered solvent water and one DMF (di­methyl­formamide) solvent mol­ecules, with occupancies of 0.5 for the water mol­ecules and of 0.25 for the DMF solvent mol­ecule. The Co—O/N bond lengths are in the range 2.094 (3)–2.216 (3) Å, comparable with those reported for other related Co^2+^-polycarboxyl­ate compounds (Gu *et al.*, 2022 ▸, 2023 ▸; Zhao *et al.*, 2024 ▸). The Co^2+^ cation is six-coordinated by four oxygen atoms from two different dmtb^2–^ anions and two nitro­gen atoms from two different dpa ligands, forming a distorted octa­hedral environment. The mean deviation of the equatorial plane constructed by atoms O1, O4*A*, O5*A* and N2 is 0.13 Å. The dmtb^2–^ ligand bridges two Co^2+^ cations in a *μ*_2_-*κ*_2_: *κ*_2_ coord­in­ation mode, so that each carboxyl­ate group of the dmtb^2–^ anion chelates one Co^2+^ cation. The dpa ligands connect the Co^2+^ cations as a ditopic linker. Accordingly, two dmtb^2–^ and two dpa ligands bridge the Co^2+^ cations into four different directions into a layered arrangement extending parallel to the *bc* plane (Fig. 2 ▸). The 5-(di­methyl­amino)­thioxometh­oxy groups dangling above and below a layer protrude into adjacent layers to display an inter­digitated motif (Fig. 3 ▸). The disordered water and DMF mol­ecules are located in the voids of this arrangement. Without these solvent mol­ecules, the void volume in (**1**) is 19.4%. The solvent mol­ecules are linked to the layers by classical hydrogen-bonding inter­actions, which includes the amino group of the dpa ligand (entries 1 and 2 in Table 1 ▸) and the water mol­ecules (entries 4–7 in Table 1 ▸) as donor groups, and the O atoms of the DMF solvent, of the water mol­ecules and the carboxyl­ate O atoms as acceptor groups. A weaker non-classical hydrogen bond between a CH group of a pyridyl ring and a carb­oxy­ate O atom consolidates the crystal packing (Fig. 4 ▸).

## Synthesis and crystallization

A mixture of Co(NO_3_)_2_·6H_2_O (29 mg, 0.1 mmol), H_2_dmtb (20 mg, 0.07 mmol) and dpa (17 mg, 0.1 mmol) in 4 ml DMF/H_2_O (*v*/*v* = 1:1) was sealed in a Teflon-lined autoclave and heated to 423 K for 72 h, then gradually cooled down to room temperature. Pink prismatic crystals were obtained. Yield: 24 mg (71%, based on H_2_dmtb).

## Refinement

Crystal data, data collection and structure refinement details are summarized in Table 2 ▸. The DMF mol­ecule was located near a symmetry center and its occupancy was fixed at 0.5. After refinement, some residual electron density peaks still existed near the DMF mol­ecule. They were assigned to the O atoms of water mol­ecules, both refined with occupancies of 0.5. ISOR and SIMU instructions in *SHELXL* (Sheldrick, 2015*b* ▸) were used for these solvent mol­ecules. Hydrogen atoms of the water mol­ecules were included in calculated positions for obtaining reasonable hydrogen bonds and were refined in a riding-model approximation with *U*_iso_(H) = 1.5_eq_(O).

## Supplementary Material

Crystal structure: contains datablock(s) I. DOI: 10.1107/S2414314624004899/wm4212sup1.cif

Structure factors: contains datablock(s) I. DOI: 10.1107/S2414314624004899/wm4212Isup3.hkl

CCDC reference: 2247034

Additional supporting information:  crystallographic information; 3D view; checkCIF report

## Figures and Tables

**Table 1:** Hydrogen-bond geometry (Å, °)

*D*—H⋯*A*	*D*—H	H⋯*A*	*D*⋯*A*	*D*—H⋯*A*
N3—H3⋯O6	0.86	1.86	2.700 (19)	164
N3—H3⋯O8	0.86	2.13	2.979 (17)	170
C13—H13⋯O2^i^	0.93	2.44	3.174 (5)	135
O7—H7*D*⋯O4	0.84	2.16	2.992 (7)	174
O7—H7*E*⋯O2^ii^	0.88	2.26	3.136 (7)	173
O8—H8*A*⋯O7^iii^	0.88	2.33	3.10 (2)	146
O8—H8*B*⋯O5^iv^	0.85	2.28	3.102 (17)	163

Symmetry codes: (i) 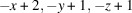; (ii) 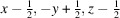; (iii) 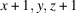; (iv) 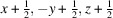.

**Table 2:** Experimental details

Crystal data	
Chemical formula	[Co(C_11_H_9_NO_5_S)(C_10_H_9_N_3_)]·0.5C_3_H_7_NO·H_2_O
*M*_r_	551.95
Crystal system, space group	Monoclinic, *P*2_1_/*n*
Temperature (K)	298
*a*, *b*, *c* (Å)	11.2451 (14), 14.4734 (17), 15.232 (2)
β (°)	103.485 (4)
*V* (Å^3^)	2410.7 (5)
*Z*	4
Radiation type	Mo *K*α
μ (mm^−1^)	0.85
Crystal size (mm)	0.32 × 0.20 × 0.18

Data collection	
Diffractometer	Bruker APEXII CCD
Absorption correction	Multi-scan *(*SADABS**; Krause *et al.*, 2015 ▸)
*T*_min_, *T*_max_	0.643, 0.745
No. of measured, independent and observed [*I* > 2σ(*I*)] reflections	21131, 4711, 3216
*R*_int_	0.069
(sin θ/λ)_max_ (Å^−1^)	0.617

Refinement	
*R*[*F*^2^ > 2σ(*F*^2^)], *wR*(*F*^2^), *S*	0.053, 0.150, 1.04
No. of reflections	4711
No. of parameters	355
No. of restraints	64
H-atom treatment	H-atom parameters constrained
Δρ_max_, Δρ_min_ (e Å^−3^)	0.76, −0.42

Computer programs: *APEX2* and *SAINT* (Bruker, 2015 ▸), *SHELXT* (Sheldrick, 2015*a* ▸), *SHELXL* (Sheldrick, 2015*b* ▸), *OLEX2* (Dolomanov *et al.*, 2009 ▸), *XP* (Sheldrick, 2008 ▸) and *publCIF* (Westrip, 2010 ▸).

## full crystallographic data

### Poly[[{µ2-5-[(dimethylamino)(thioxo)methoxy]benzene-1,3-dicarboxylato-κ4O1,O1':O3,O3'}(µ2-4,4'-dipyridylamine-κ2N4:N4')cobalt(II)] dimethylformamide hemisolvate monohydrate] Crystal data

**Table:**

[Co(C_11_H_9_NO_5_S)(C_10_H_9_N_3_)]·0.5C_3_H_7_NO·H_2_O	*F*(000) = 1140
*M**_r_* = 551.95	*D*_x_ = 1.521 Mg m^−^^3^
Monoclinic, *P*2_1_/*n*	Mo *K*α radiation, λ = 0.71073 Å
*a* = 11.2451 (14) Å	Cell parameters from 4852 reflections
*b* = 14.4734 (17) Å	θ = 2.9–26.3°
*c* = 15.232 (2) Å	µ = 0.85 mm^−^^1^
β = 103.485 (4)°	*T* = 298 K
*V* = 2410.7 (5) Å^3^	Prism, purple
*Z* = 4	0.32 × 0.20 × 0.18 mm

### Poly[[{µ2-5-[(dimethylamino)(thioxo)methoxy]benzene-1,3-dicarboxylato-κ4O1,O1':O3,O3'}(µ2-4,4'-dipyridylamine-κ2N4:N4')cobalt(II)] dimethylformamide hemisolvate monohydrate] Data collection

**Table:**

Bruker APEXII CCD diffractometer	3216 reflections with *I* > 2σ(*I*)
φ and ω scans	*R*_int_ = 0.069
Absorption correction: multi-scan *(SADABS*; Krause *et al.*, 2015)	θ_max_ = 26.0°, θ_min_ = 2.5°
*T*_min_ = 0.643, *T*_max_ = 0.745	*h* = −13→13
21131 measured reflections	*k* = −17→17
4711 independent reflections	*l* = −18→18

### Poly[[{µ2-5-[(dimethylamino)(thioxo)methoxy]benzene-1,3-dicarboxylato-κ4O1,O1':O3,O3'}(µ2-4,4'-dipyridylamine-κ2N4:N4')cobalt(II)] dimethylformamide hemisolvate monohydrate] Refinement

**Table:**

Refinement on *F*^2^	Primary atom site location: dual
Least-squares matrix: full	Hydrogen site location: mixed
*R*[*F*^2^ > 2σ(*F*^2^)] = 0.053	H-atom parameters constrained
*wR*(*F*^2^) = 0.150	*w* = 1/[σ^2^(*F*_o_^2^) + (0.0742*P*)^2^ + 2.1129*P*] where *P* = (*F*_o_^2^ + 2*F*_c_^2^)/3
*S* = 1.04	(Δ/σ)_max_ = 0.001
4711 reflections	Δρ_max_ = 0.76 e Å^−^^3^
355 parameters	Δρ_min_ = −0.42 e Å^−^^3^
64 restraints	

### Poly[[{µ2-5-[(dimethylamino)(thioxo)methoxy]benzene-1,3-dicarboxylato-κ4O1,O1':O3,O3'}(µ2-4,4'-dipyridylamine-κ2N4:N4')cobalt(II)] dimethylformamide hemisolvate monohydrate] Special details

**Table:**

Geometry. All esds (except the esd in the dihedral angle between two l.s. planes) are estimated using the full covariance matrix. The cell esds are taken into account individually in the estimation of esds in distances, angles and torsion angles; correlations between esds in cell parameters are only used when they are defined by crystal symmetry. An approximate (isotropic) treatment of cell esds is used for estimating esds involving l.s. planes.

### Poly[[{µ2-5-[(dimethylamino)(thioxo)methoxy]benzene-1,3-dicarboxylato-κ4O1,O1':O3,O3'}(µ2-4,4'-dipyridylamine-κ2N4:N4')cobalt(II)] dimethylformamide hemisolvate monohydrate] Fractional atomic coordinates and isotropic or equivalent isotropic displacement parameters (Å^2^)

**Table:**

	*x*	*y*	*z*	*U*_iso_*/*U*_eq_	Occ. (<1)
Co1	0.73927 (5)	0.60696 (3)	0.44875 (3)	0.03359 (18)	
S1	0.21293 (14)	0.44589 (10)	0.06254 (10)	0.0733 (4)	
O1	0.5793 (2)	0.53436 (19)	0.38364 (19)	0.0461 (7)	
O2	0.7529 (3)	0.4927 (2)	0.3548 (2)	0.0535 (8)	
O3	0.2652 (2)	0.32239 (19)	0.19296 (19)	0.0456 (7)	
O4	0.5950 (3)	0.18067 (18)	0.05399 (18)	0.0460 (7)	
O5	0.7602 (3)	0.2134 (2)	0.15532 (19)	0.0510 (8)	
N1	0.0682 (3)	0.3554 (2)	0.1501 (2)	0.0469 (9)	
N2	0.8119 (3)	0.5239 (2)	0.5610 (2)	0.0372 (7)	
N3	0.9644 (3)	0.3791 (2)	0.8000 (2)	0.0412 (8)	
H3	1.042657	0.385181	0.814726	0.049*	
N4	0.8350 (3)	0.2025 (2)	0.9750 (2)	0.0393 (8)	
C1	0.6393 (4)	0.4844 (2)	0.3406 (2)	0.0349 (8)	
C2	0.5745 (3)	0.4151 (2)	0.2733 (2)	0.0327 (8)	
C3	0.4479 (4)	0.4062 (2)	0.2574 (3)	0.0366 (9)	
H3A	0.403355	0.444495	0.286860	0.044*	
C4	0.3893 (3)	0.3402 (3)	0.1975 (3)	0.0372 (9)	
C5	0.1790 (4)	0.3741 (3)	0.1362 (3)	0.0440 (10)	
C6	−0.0385 (4)	0.4035 (3)	0.0981 (3)	0.0567 (12)	
H6A	−0.061594	0.451505	0.134299	0.085*	
H6B	−0.104911	0.360578	0.080358	0.085*	
H6C	−0.019640	0.430104	0.045232	0.085*	
C7	0.0470 (4)	0.2900 (4)	0.2157 (4)	0.0664 (14)	
H7A	0.071609	0.229571	0.200946	0.100*	
H7B	−0.038419	0.289331	0.215602	0.100*	
H7C	0.093632	0.307415	0.274533	0.100*	
C8	0.4527 (4)	0.2843 (3)	0.1506 (3)	0.0376 (9)	
H8	0.411067	0.242145	0.108353	0.045*	
C9	0.5783 (3)	0.2916 (2)	0.1671 (2)	0.0331 (8)	
C10	0.6488 (4)	0.2256 (2)	0.1232 (2)	0.0359 (9)	
C11	0.6394 (3)	0.3569 (2)	0.2289 (2)	0.0328 (8)	
H11	0.724132	0.361467	0.240303	0.039*	
C12	0.9316 (4)	0.5240 (3)	0.6018 (3)	0.0432 (10)	
H12	0.983755	0.557585	0.574663	0.052*	
C13	0.9820 (4)	0.4786 (3)	0.6798 (3)	0.0419 (9)	
H13	1.065719	0.482183	0.704535	0.050*	
C14	0.9069 (4)	0.4261 (2)	0.7228 (2)	0.0359 (9)	
C15	0.9165 (4)	0.3234 (2)	0.8576 (2)	0.0374 (9)	
C16	0.7984 (4)	0.3275 (3)	0.8688 (2)	0.0418 (9)	
H16	0.743752	0.371028	0.837599	0.050*	
C17	0.7625 (4)	0.2660 (3)	0.9269 (2)	0.0418 (9)	
H17	0.682131	0.269181	0.932704	0.050*	
C18	0.9509 (4)	0.2020 (3)	0.9670 (3)	0.0459 (10)	
H18	1.004311	0.159659	1.001410	0.055*	
C19	0.9959 (4)	0.2596 (3)	0.9116 (3)	0.0441 (10)	
H19	1.077847	0.256815	0.909575	0.053*	
C20	0.7837 (4)	0.4238 (3)	0.6808 (3)	0.0439 (10)	
H20	0.729663	0.389357	0.705490	0.053*	
C21	0.7419 (4)	0.4728 (3)	0.6025 (3)	0.0414 (9)	
H21	0.658501	0.470415	0.576267	0.050*	
O6	1.2102 (17)	0.3656 (14)	0.8304 (14)	0.116 (6)	0.5
O7	0.3470 (7)	0.2021 (5)	−0.0692 (5)	0.090 (3)	0.5
H7D	0.414831	0.198915	−0.032112	0.135*	0.5
H7E	0.317801	0.146795	−0.086502	0.135*	0.5
C23	1.4935 (17)	0.4546 (13)	0.9452 (13)	0.199 (8)	0.5
H23A	1.466619	0.517120	0.949026	0.298*	0.5
H23B	1.561309	0.453770	0.916886	0.298*	0.5
H23C	1.518269	0.428940	1.004746	0.298*	0.5
N5	1.4060 (15)	0.4024 (12)	0.8994 (13)	0.189 (6)	0.5
C22	1.2767 (15)	0.4296 (13)	0.8683 (16)	0.166 (6)	0.5
H22	1.247808	0.488525	0.876167	0.199*	0.5
C24	1.424 (2)	0.3087 (13)	0.8792 (19)	0.237 (9)	0.5
H24A	1.509290	0.297487	0.884281	0.356*	0.5
H24B	1.379160	0.295245	0.818744	0.356*	0.5
H24C	1.394999	0.269661	0.920830	0.356*	0.5
O8	1.2362 (15)	0.3812 (15)	0.8331 (9)	0.109 (6)	0.5
H8A	1.283550	0.349024	0.876365	0.164*	0.5
H8B	1.255520	0.363564	0.785015	0.164*	0.5

### Poly[[{µ2-5-[(dimethylamino)(thioxo)methoxy]benzene-1,3-dicarboxylato-κ4O1,O1':O3,O3'}(µ2-4,4'-dipyridylamine-κ2N4:N4')cobalt(II)] dimethylformamide hemisolvate monohydrate] Atomic displacement parameters (Å^2^)

**Table:**

	*U*^11^	*U*^22^	*U*^33^	*U*^12^	*U*^13^	*U*^23^
Co1	0.0496 (3)	0.0268 (3)	0.0268 (3)	−0.0025 (2)	0.0137 (2)	−0.0018 (2)
S1	0.0705 (9)	0.0671 (9)	0.0750 (9)	0.0037 (7)	0.0023 (7)	0.0295 (7)
O1	0.0447 (16)	0.0435 (16)	0.0524 (17)	−0.0023 (13)	0.0158 (14)	−0.0207 (13)
O2	0.0431 (18)	0.071 (2)	0.0508 (18)	−0.0167 (15)	0.0189 (14)	−0.0265 (15)
O3	0.0296 (15)	0.0517 (16)	0.0523 (17)	0.0015 (12)	0.0033 (13)	0.0137 (13)
O4	0.0552 (18)	0.0390 (15)	0.0452 (16)	−0.0038 (13)	0.0146 (14)	−0.0154 (13)
O5	0.0505 (19)	0.0645 (19)	0.0398 (16)	0.0128 (15)	0.0140 (14)	−0.0109 (14)
N1	0.035 (2)	0.049 (2)	0.049 (2)	0.0063 (16)	−0.0050 (16)	−0.0034 (17)
N2	0.053 (2)	0.0305 (16)	0.0321 (16)	−0.0019 (15)	0.0178 (15)	0.0024 (13)
N3	0.055 (2)	0.0375 (18)	0.0328 (17)	−0.0046 (15)	0.0133 (15)	0.0085 (14)
N4	0.062 (2)	0.0301 (16)	0.0278 (16)	−0.0028 (15)	0.0155 (15)	0.0016 (13)
C1	0.044 (2)	0.0323 (19)	0.0303 (19)	−0.0057 (17)	0.0131 (17)	−0.0028 (15)
C2	0.035 (2)	0.0322 (19)	0.0317 (19)	−0.0007 (15)	0.0100 (16)	−0.0002 (15)
C3	0.040 (2)	0.033 (2)	0.039 (2)	0.0070 (16)	0.0119 (17)	−0.0028 (16)
C4	0.034 (2)	0.039 (2)	0.037 (2)	0.0013 (17)	0.0052 (17)	0.0078 (17)
C5	0.046 (3)	0.037 (2)	0.040 (2)	0.0086 (18)	−0.0076 (19)	−0.0092 (17)
C6	0.043 (3)	0.057 (3)	0.059 (3)	0.015 (2)	−0.010 (2)	−0.011 (2)
C7	0.045 (3)	0.078 (3)	0.076 (3)	0.000 (2)	0.013 (2)	0.015 (3)
C8	0.042 (2)	0.034 (2)	0.034 (2)	−0.0048 (17)	0.0021 (17)	−0.0014 (16)
C9	0.039 (2)	0.0337 (19)	0.0284 (18)	−0.0011 (16)	0.0120 (16)	−0.0033 (15)
C10	0.046 (2)	0.0316 (19)	0.033 (2)	−0.0005 (17)	0.0148 (18)	0.0021 (16)
C11	0.032 (2)	0.0368 (19)	0.0307 (19)	−0.0009 (16)	0.0091 (15)	−0.0024 (16)
C12	0.052 (3)	0.042 (2)	0.043 (2)	−0.0035 (19)	0.025 (2)	0.0106 (18)
C13	0.046 (2)	0.043 (2)	0.042 (2)	−0.0049 (18)	0.0210 (19)	0.0059 (18)
C14	0.053 (2)	0.0265 (18)	0.0313 (19)	0.0003 (17)	0.0167 (18)	−0.0002 (15)
C15	0.058 (3)	0.0304 (19)	0.0250 (18)	−0.0051 (18)	0.0122 (17)	−0.0012 (15)
C16	0.059 (3)	0.036 (2)	0.031 (2)	−0.0003 (19)	0.0096 (18)	0.0046 (16)
C17	0.051 (3)	0.040 (2)	0.034 (2)	−0.0045 (19)	0.0088 (18)	0.0073 (17)
C18	0.066 (3)	0.034 (2)	0.040 (2)	0.015 (2)	0.018 (2)	0.0077 (17)
C19	0.057 (3)	0.041 (2)	0.040 (2)	0.007 (2)	0.022 (2)	0.0036 (18)
C20	0.058 (3)	0.041 (2)	0.036 (2)	−0.014 (2)	0.0167 (19)	0.0061 (17)
C21	0.049 (2)	0.039 (2)	0.036 (2)	−0.0113 (18)	0.0098 (18)	0.0028 (17)
O6	0.134 (10)	0.108 (8)	0.110 (9)	0.001 (7)	0.031 (7)	−0.003 (6)
O7	0.085 (6)	0.075 (5)	0.084 (5)	0.023 (4)	−0.034 (4)	−0.032 (4)
C23	0.166 (13)	0.206 (13)	0.211 (14)	−0.064 (11)	0.018 (11)	0.087 (11)
N5	0.190 (8)	0.193 (8)	0.179 (8)	0.003 (7)	0.031 (7)	0.030 (7)
C22	0.168 (9)	0.169 (8)	0.161 (8)	0.002 (7)	0.040 (7)	0.020 (7)
C24	0.233 (14)	0.249 (15)	0.219 (14)	0.067 (13)	0.034 (12)	−0.036 (13)
O8	0.100 (9)	0.192 (16)	0.034 (5)	−0.055 (9)	0.013 (5)	0.020 (7)

### Poly[[{µ2-5-[(dimethylamino)(thioxo)methoxy]benzene-1,3-dicarboxylato-κ4O1,O1':O3,O3'}(µ2-4,4'-dipyridylamine-κ2N4:N4')cobalt(II)] dimethylformamide hemisolvate monohydrate] Geometric parameters (Å, º)

**Table:**

Co1—O1	2.119 (3)	C8—C9	1.380 (5)
Co1—O2	2.216 (3)	C9—C10	1.496 (5)
Co1—O4^i^	2.156 (3)	C9—C11	1.395 (5)
Co1—O5^i^	2.211 (3)	C11—H11	0.9300
Co1—N2	2.094 (3)	C12—H12	0.9300
Co1—N4^ii^	2.100 (3)	C12—C13	1.361 (5)
Co1—C1	2.502 (4)	C13—H13	0.9300
S1—C5	1.638 (5)	C13—C14	1.406 (5)
O1—C1	1.271 (4)	C14—C20	1.385 (6)
O2—C1	1.251 (5)	C15—C16	1.378 (6)
O3—C4	1.404 (4)	C15—C19	1.409 (5)
O3—C5	1.363 (4)	C16—H16	0.9300
O4—C10	1.266 (4)	C16—C17	1.380 (5)
O5—C10	1.247 (5)	C17—H17	0.9300
N1—C5	1.339 (6)	C18—H18	0.9300
N1—C6	1.451 (5)	C18—C19	1.364 (6)
N1—C7	1.437 (6)	C19—H19	0.9300
N2—C12	1.345 (5)	C20—H20	0.9300
N2—C21	1.341 (5)	C20—C21	1.373 (5)
N3—H3	0.8600	C21—H21	0.9300
N3—C14	1.382 (5)	O6—C22	1.245 (17)
N3—C15	1.390 (5)	O7—H7D	0.8380
N4—C17	1.330 (5)	O7—H7E	0.8812
N4—C18	1.337 (5)	C23—H23A	0.9598
C1—C2	1.496 (5)	C23—H23B	0.9600
C2—C3	1.393 (5)	C23—H23C	0.9595
C2—C11	1.390 (5)	C23—N5	1.306 (14)
C3—H3A	0.9300	N5—C22	1.474 (15)
C3—C4	1.379 (5)	N5—C24	1.416 (15)
C4—C8	1.381 (5)	C22—H22	0.9300
C6—H6A	0.9600	C22—O8	0.93 (3)
C6—H6B	0.9600	C24—H24A	0.9600
C6—H6C	0.9600	C24—H24B	0.9600
C7—H7A	0.9600	C24—H24C	0.9600
C7—H7B	0.9600	O8—H8A	0.8784
C7—H7C	0.9600	O8—H8B	0.8500
C8—H8	0.9300		
			
O1—Co1—O2	60.43 (10)	C4—C8—H8	120.3
O1—Co1—O4^i^	151.57 (11)	C9—C8—C4	119.4 (3)
O1—Co1—O5^i^	98.85 (11)	C9—C8—H8	120.3
O1—Co1—C1	30.50 (11)	C8—C9—C10	119.8 (3)
O2—Co1—C1	29.96 (11)	C8—C9—C11	119.8 (3)
O4^i^—Co1—O2	99.60 (11)	C11—C9—C10	120.3 (3)
O4^i^—Co1—O5^i^	59.65 (10)	O4—C10—C9	120.0 (4)
O4^i^—Co1—C1	126.57 (12)	O5—C10—O4	119.7 (4)
O5^i^—Co1—O2	92.57 (12)	O5—C10—C9	120.3 (3)
O5^i^—Co1—C1	95.62 (11)	C2—C11—C9	120.4 (3)
N2—Co1—O1	102.95 (12)	C2—C11—H11	119.8
N2—Co1—O2	91.51 (12)	C9—C11—H11	119.8
N2—Co1—O4^i^	97.23 (12)	N2—C12—H12	117.6
N2—Co1—O5^i^	156.87 (12)	N2—C12—C13	124.9 (4)
N2—Co1—N4^ii^	93.24 (12)	C13—C12—H12	117.6
N2—Co1—C1	99.22 (12)	C12—C13—H13	120.2
N4^ii^—Co1—O1	100.46 (12)	C12—C13—C14	119.6 (4)
N4^ii^—Co1—O2	160.89 (13)	C14—C13—H13	120.2
N4^ii^—Co1—O4^i^	98.15 (12)	N3—C14—C13	116.7 (4)
N4^ii^—Co1—O5^i^	90.30 (11)	N3—C14—C20	126.9 (4)
N4^ii^—Co1—C1	130.93 (14)	C20—C14—C13	116.3 (4)
C1—O1—Co1	91.7 (2)	N3—C15—C19	117.5 (4)
C1—O2—Co1	87.8 (2)	C16—C15—N3	125.5 (4)
C5—O3—C4	118.8 (3)	C16—C15—C19	117.0 (3)
C10—O4—Co1^iii^	91.3 (2)	C15—C16—H16	120.5
C10—O5—Co1^iii^	89.3 (2)	C15—C16—C17	119.1 (4)
C5—N1—C6	119.9 (4)	C17—C16—H16	120.5
C5—N1—C7	123.6 (3)	N4—C17—C16	124.5 (4)
C7—N1—C6	116.4 (4)	N4—C17—H17	117.8
C12—N2—Co1	122.3 (2)	C16—C17—H17	117.8
C21—N2—Co1	122.8 (3)	N4—C18—H18	117.9
C21—N2—C12	114.6 (3)	N4—C18—C19	124.3 (4)
C14—N3—H3	114.8	C19—C18—H18	117.9
C14—N3—C15	130.5 (4)	C15—C19—H19	120.5
C15—N3—H3	114.8	C18—C19—C15	119.0 (4)
C17—N4—Co1^iv^	119.2 (3)	C18—C19—H19	120.5
C17—N4—C18	116.0 (3)	C14—C20—H20	120.3
C18—N4—Co1^iv^	124.7 (3)	C21—C20—C14	119.5 (4)
O1—C1—Co1	57.84 (18)	C21—C20—H20	120.3
O1—C1—C2	120.2 (3)	N2—C21—C20	125.1 (4)
O2—C1—Co1	62.2 (2)	N2—C21—H21	117.5
O2—C1—O1	120.0 (3)	C20—C21—H21	117.5
O2—C1—C2	119.8 (3)	H7D—O7—H7E	111.7
C2—C1—Co1	176.8 (3)	H23A—C23—H23B	109.5
C3—C2—C1	119.7 (3)	H23A—C23—H23C	109.5
C11—C2—C1	120.9 (3)	H23B—C23—H23C	109.5
C11—C2—C3	119.4 (3)	N5—C23—H23A	111.7
C2—C3—H3A	120.3	N5—C23—H23B	109.3
C4—C3—C2	119.4 (3)	N5—C23—H23C	107.3
C4—C3—H3A	120.3	C23—N5—C22	125.5 (15)
C3—C4—O3	118.4 (3)	C23—N5—C24	122.9 (15)
C3—C4—C8	121.6 (4)	C24—N5—C22	111.4 (13)
C8—C4—O3	119.6 (3)	O6—C22—N5	113.0 (15)
O3—C5—S1	122.5 (3)	O6—C22—H22	123.5
N1—C5—S1	127.6 (3)	N5—C22—H22	123.5
N1—C5—O3	109.9 (4)	O8—C22—O6	10.1 (19)
N1—C6—H6A	109.5	O8—C22—N5	107 (2)
N1—C6—H6B	109.5	O8—C22—H22	128.6
N1—C6—H6C	109.5	N5—C24—H24A	109.5
H6A—C6—H6B	109.5	N5—C24—H24B	109.5
H6A—C6—H6C	109.5	N5—C24—H24C	109.5
H6B—C6—H6C	109.5	H24A—C24—H24B	109.5
N1—C7—H7A	109.5	H24A—C24—H24C	109.5
N1—C7—H7B	109.5	H24B—C24—H24C	109.5
N1—C7—H7C	109.5	C22—O8—H8A	80.7
H7A—C7—H7B	109.5	C22—O8—H8B	122.1
H7A—C7—H7C	109.5	H8A—O8—H8B	104.8
H7B—C7—H7C	109.5		
			
Co1—O1—C1—O2	3.4 (4)	C5—O3—C4—C3	87.8 (4)
Co1—O1—C1—C2	−177.0 (3)	C5—O3—C4—C8	−100.0 (4)
Co1—O2—C1—O1	−3.2 (4)	C6—N1—C5—S1	−1.8 (6)
Co1—O2—C1—C2	177.2 (3)	C6—N1—C5—O3	179.0 (3)
Co1^iii^—O4—C10—O5	2.2 (4)	C7—N1—C5—S1	179.1 (4)
Co1^iii^—O4—C10—C9	−176.6 (3)	C7—N1—C5—O3	−0.1 (6)
Co1^iii^—O5—C10—O4	−2.1 (4)	C8—C9—C10—O4	19.2 (5)
Co1^iii^—O5—C10—C9	176.7 (3)	C8—C9—C10—O5	−159.6 (4)
Co1—N2—C12—C13	173.3 (3)	C8—C9—C11—C2	−0.5 (5)
Co1—N2—C21—C20	−173.9 (3)	C10—C9—C11—C2	−176.6 (3)
Co1^iv^—N4—C17—C16	−174.6 (3)	C11—C2—C3—C4	−0.3 (5)
Co1^iv^—N4—C18—C19	174.4 (3)	C11—C9—C10—O4	−164.7 (3)
O1—C1—C2—C3	0.7 (5)	C11—C9—C10—O5	16.5 (5)
O1—C1—C2—C11	−176.9 (3)	C12—N2—C21—C20	0.5 (6)
O2—C1—C2—C3	−179.7 (4)	C12—C13—C14—N3	177.7 (4)
O2—C1—C2—C11	2.7 (5)	C12—C13—C14—C20	0.6 (6)
O3—C4—C8—C9	−169.0 (3)	C13—C14—C20—C21	−1.2 (6)
N2—C12—C13—C14	0.6 (6)	C14—N3—C15—C16	−23.9 (6)
N3—C14—C20—C21	−178.0 (4)	C14—N3—C15—C19	158.4 (4)
N3—C15—C16—C17	178.3 (4)	C14—C20—C21—N2	0.6 (6)
N3—C15—C19—C18	−178.0 (4)	C15—N3—C14—C13	178.9 (4)
N4—C18—C19—C15	−1.1 (6)	C15—N3—C14—C20	−4.3 (6)
C1—C2—C3—C4	−177.9 (3)	C15—C16—C17—N4	1.0 (6)
C1—C2—C11—C9	179.1 (3)	C16—C15—C19—C18	4.1 (6)
C2—C3—C4—O3	170.1 (3)	C17—N4—C18—C19	−2.0 (6)
C2—C3—C4—C8	−2.0 (6)	C18—N4—C17—C16	2.0 (6)
C3—C2—C11—C9	1.5 (5)	C19—C15—C16—C17	−4.0 (5)
C3—C4—C8—C9	3.1 (6)	C21—N2—C12—C13	−1.2 (6)
C4—O3—C5—S1	8.9 (5)	C23—N5—C22—O6	−176 (2)
C4—O3—C5—N1	−171.9 (3)	C23—N5—C22—O8	175 (2)
C4—C8—C9—C10	174.4 (3)	C24—N5—C22—O6	−1 (3)
C4—C8—C9—C11	−1.8 (5)	C24—N5—C22—O8	−9 (3)

Symmetry codes: (i) −*x*+3/2, *y*+1/2, −*z*+1/2; (ii) −*x*+3/2, *y*+1/2, −*z*+3/2; (iii) −*x*+3/2, *y*−1/2, −*z*+1/2; (iv) −*x*+3/2, *y*−1/2, −*z*+3/2.

### Poly[[{µ2-5-[(dimethylamino)(thioxo)methoxy]benzene-1,3-dicarboxylato-κ4O1,O1':O3,O3'}(µ2-4,4'-dipyridylamine-κ2N4:N4')cobalt(II)] dimethylformamide hemisolvate monohydrate] Hydrogen-bond geometry (Å, º)

**Table:**

*D*—H···*A*	*D*—H	H···*A*	*D*···*A*	*D*—H···*A*
N3—H3···O6	0.86	1.86	2.700 (19)	164
N3—H3···O8	0.86	2.13	2.979 (17)	170
C13—H13···O2^v^	0.93	2.44	3.174 (5)	135
O7—H7*D*···O4	0.84	2.16	2.992 (7)	174
O7—H7*E*···O2^vi^	0.88	2.26	3.136 (7)	173
O8—H8*A*···O7^vii^	0.88	2.33	3.10 (2)	146
O8—H8*B*···O5^viii^	0.85	2.28	3.102 (17)	163

Symmetry codes: (v) −*x*+2, −*y*+1, −*z*+1; (vi) *x*−1/2, −*y*+1/2, *z*−1/2; (vii) *x*+1, *y*, *z*+1; (viii) *x*+1/2, −*y*+1/2, *z*+1/2.
